# A phase-II/III randomized controlled trial of adjuvant radiotherapy or concurrent chemoradiotherapy after surgery versus surgery alone in patients with stage-IIB/III esophageal squamous cell carcinoma

**DOI:** 10.1186/s12885-020-6592-2

**Published:** 2020-02-18

**Authors:** Wenjie Ni, Shufei Yu, Wencheng Zhang, Zefen Xiao, Zongmei Zhou, Dongfu Chen, Qinfu Feng, Jun Liang, Jima Lv, Shugeng Gao, Yousheng Mao, Qi Xue, Kelin Sun, Xiangyang Liu, Dekang Fang, Jian Li, Dali Wang

**Affiliations:** 1 Department of Radiation Oncology, National Cancer Center/National Clinical Research Center for Cancer/Cancer Hospital, Chinese Academy of Medical Sciences and Peking Union Medical College, No. 17 South Panjiayuan lane, Chaoyang District, Beijing, 100021 China; 2 Department of Radiotherapy, Beijing Chao-yang Hospital, Capital Medical University, Beijing, China; 3 Department of Radiation Oncology, Tianjing Medical University Cancer Institute and Hospital, National Clinical Research Center of Cancer, Tianjin, China; 4 Department of Thoracic Surgery, National Cancer Center/National Clinical Research Center for Cancer/Cancer Hospital, Chinese Academy of Medical Sciences and Peking Union Medical College, Beijing, China

**Keywords:** Esophageal cancer, Adjuvant therapy, Chemoradiotherapy, Surgery

## Abstract

**Background:**

Preoperative chemoradiotherapy (CRT) followed by surgery is the most common approach for patients with resectable esophageal cancer. Nevertheless, considerable numbers of esophageal-cancer patients undergo surgery as the first treatment. The benefit of neoadjuvant therapy might only be for patients with a pathologic complete response, so stratified research is necessary. Postoperative treatments have important roles because of the poor survival rates of patients with stage-IIB/III disease treated with resection alone. Five-year survival of patients with stage-IIB/III thoracic esophageal squamous cell carcinoma (TESCC) after surgery is 20.0–28.4%, and locoregional lymph-node metastases are the main cause of failure. Several retrospective studies have shown that postoperative radiotherapy (PORT) and postoperative chemoradiotherapy (POCRT) after radical esophagectomy for esophageal carcinoma with positive lymph-node metastases and stage-III disease can decrease locoregional recurrence and increase overall survival (OS). Using intensity-modulated RT, PORT reduces locoregional recurrence further. However, the rate of distant metastases increases to 30.7%. Hence, chemotherapy may be vital for these patients. Therefore, a prospective randomized controlled trial (RCT) is needed to evaluate the value of PORT and concurrent POCRT in comparison with surgery alone (SA) for esophageal cancer.

**Method:**

This will be a phase-II/III RCT. The patients with pathologic stage-IIB/III esophageal squamous cell carcinoma will receive concurrent POCRT or PORT after radical esophagectomy compared with those who have SA. A total of 120 patients in each group will be recruited. POCRT patients will be 50.4 Gy concurrent with paclitaxel (135–150 mg/m^2^) plus cisplatin or nedaplatin (50–75 mg/m^2^) treatment every 28 days. Two cycles will be required for concurrent chemotherapy. The prescription dose will be 54 Gy for PORT. The primary endpoint will be disease-free survival (DFS). The secondary endpoint will be OS. Other pre-specified outcome measures will be the proportion of patients who complete treatment, toxicity, and out-of-field regional recurrence rate between PORT and POCRT.

**Discussion:**

This prospective RCT will provide high-level evidence for postoperative adjuvant treatment of pathologic stage-IIB/III esophageal squamous cell carcinoma.

**Trial registration:**

clinicaltrials.gov (NCT02279134). Registered on October 26, 2014.

## Background

The number of cases of esophageal cancer per year in China is ~ 480,000 [1]. Locoregional recurrence occurs in 23.8–58.0% of people after radical resection of thoracic esophageal squamous cell carcinoma (TESCC) [2–6]. Moreover, spread to mediastinal lymph nodes and bilateral supraclavicular lymph nodes can occur [7, 8].

Postoperative radiotherapy (PORT) was applied first to treatment of esophageal cancer in 1969 [9]. Whether PORT can improve the overall survival (OS) for esophageal cancer is controversial [10–14]. Three main issues have arisen from perspective studies. First, identifying patients who may benefit from PORT is difficult because studies conducted so far have involved small study cohorts. Second, obtaining consistent results is challenging because radiation fields and radiation doses differ between studies. Third, Fox and colleagues [11] showed severe toxicity related to PORT, which might be associated with a high dose per fraction.

Several large retrospective studies have shown that PORT or concurrent postoperative chemoradiotherapy (POCRT) after radical esophagectomy for esophageal carcinoma with positive lymph-node metastases and stage-III disease can increase OS [15–23]. However, the rate of distant metastasis increases after PORT. However, a prospective randomized controlled trial (RCT) to ascertain if POCRT can decrease the rate of hematogenous metastasis has not been conducted.

A prospective phase-I clinical trial to compare the effect between POCRT or PORT after esophagectomy has been completed at our institution. Thus, a phase-II/III RCT in these patients is warranted to explore the safety and efficacy of adjuvant treatment.

## Methods

### Study hypotheses

The study hypotheses are that: (i) the adjuvant-treatment group (PORT/POCRT) can increase the disease-free survival (DFS) rate compared with the surgery alone (SA) group; (ii) a reduction in the radiation field using POCRT will not increase the out-of-field regional recurrence rate (OoFRRR) compared with that using PORT.

### Study design

This will be a prospective phase-II/III RCT to compare the effect between POCRT or PORT and SA after esophagectomy from October 2014 to December 2019. We have calculated that 120 patients in each group will be needed. The primary endpoint will be DFS. The secondary endpoint will be OS. Other pre-specified outcome measures will be the proportion of people who complete treatment, toxicity, and the OoFRRR between the two adjuvant-treatment groups. If the DFS rate of the adjuvant treatment groups is significantly different from that of the SA group, the latter group will be closed out due to ethical considerations. Then, the RCT will be between POCRT and PORT.

### Inclusion criteria

The inclusion criteria will be patients: (i) aged 18–68 years with pathologically proven stage-IIB or -III (as defined by Union for International Cancer Control (UICC) guidelines, 7th edition) esophageal squamous cell carcinoma undergoing radical resection (R0) with no other treatment before recruitment; (ii) Karnofsky Performance Status score ≥ 70; (iii) normal blood data; normal biochemistry data; (iv) no local regional recurrence or distant metastasis after surgery and before recruitment in our hospital; (v) who can undergo intensity-modulated radiotherapy (IMRT) or volumetric modulated arc therapy; (vi) who can undergo regular reexamination after treatment.

### Exclusion criteria

Patients will be excluded if: (i) they have uncontrolled diabetes mellitus; (ii) the interval between the surgical procedure and adjuvant therapy is > 3 months; (iii) they show signs of recurrence on computed tomography (CT), ultrasound or positron emission tomography–CT; (iv) suffer weight loss > 10% from baseline; (v) they have a concurrent malignancy or had a malignancy within 5 years other than basal cell skin cancer or carcinoma in situ of the cervix; (vi) are pregnant.

### Treatment

#### PORT

**Fig. 1:**
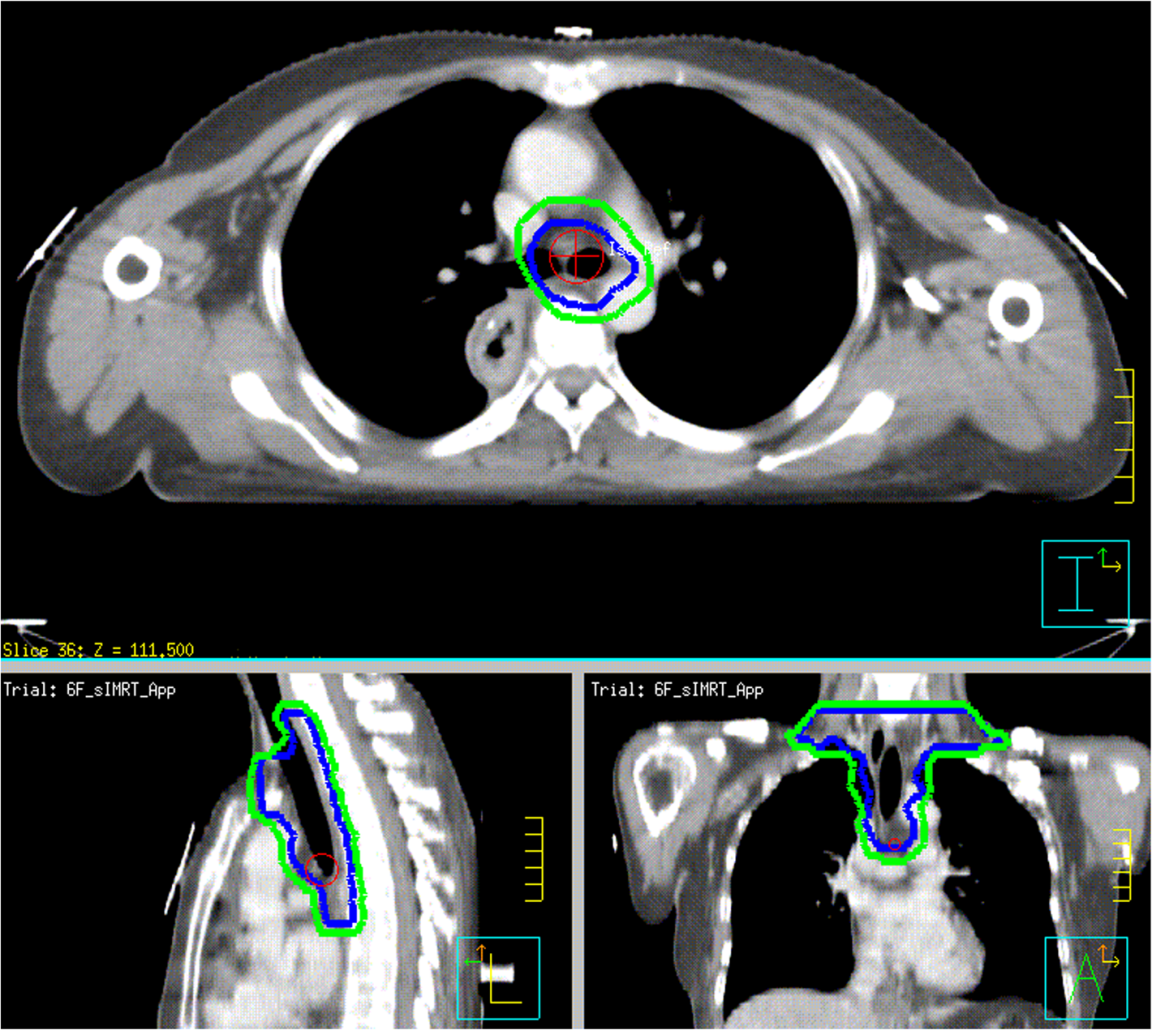
Upper-thoracic esophagus (upper margin: cricothyroid membrane; lower margin: 3 cm inferior to the lower margin of the tumor bed or subcarina; lymph-node stations include the lower cervical and bilateral supraclavicular stations 1R, 1 L, 2R, 2 L, 3p, 4R, 4 L, and 7)

**Fig. 2:**
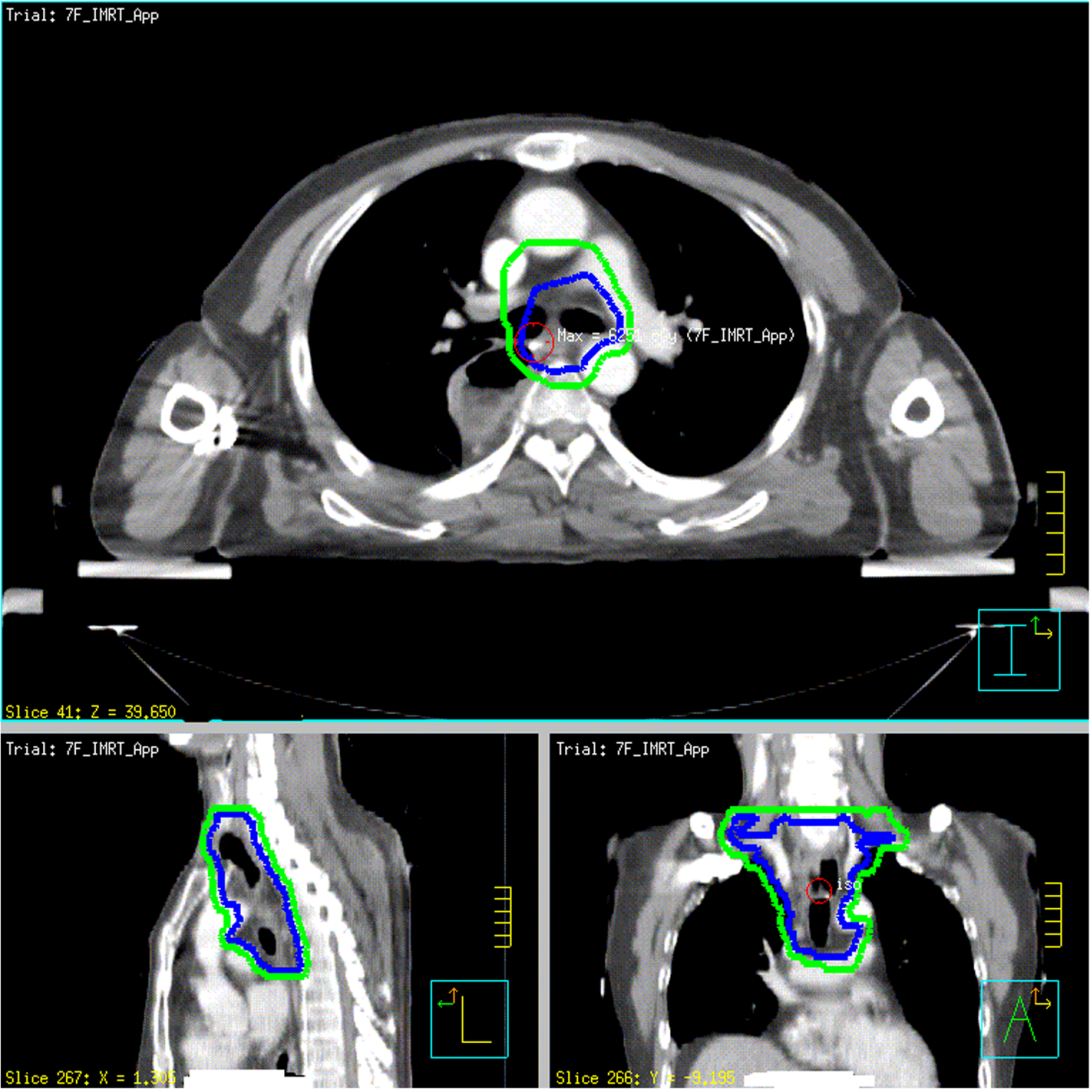
Middle-thoracic esophagus with metastasis in 0 to 2 regional lymph nodes or metastasis in ≥3 regional lymph nodes in the mediastinum (upper margin of the first thoracic vertebral body; lower margin: 3 cm below the lower border of the tumor bed; lymph-node stations include the lower cervical and bilateral supraclavicular stations 1R, 1 L, 2R, 2 L, 3p, 4R, 4 L, 7, part of 8)

**Fig. 3:**
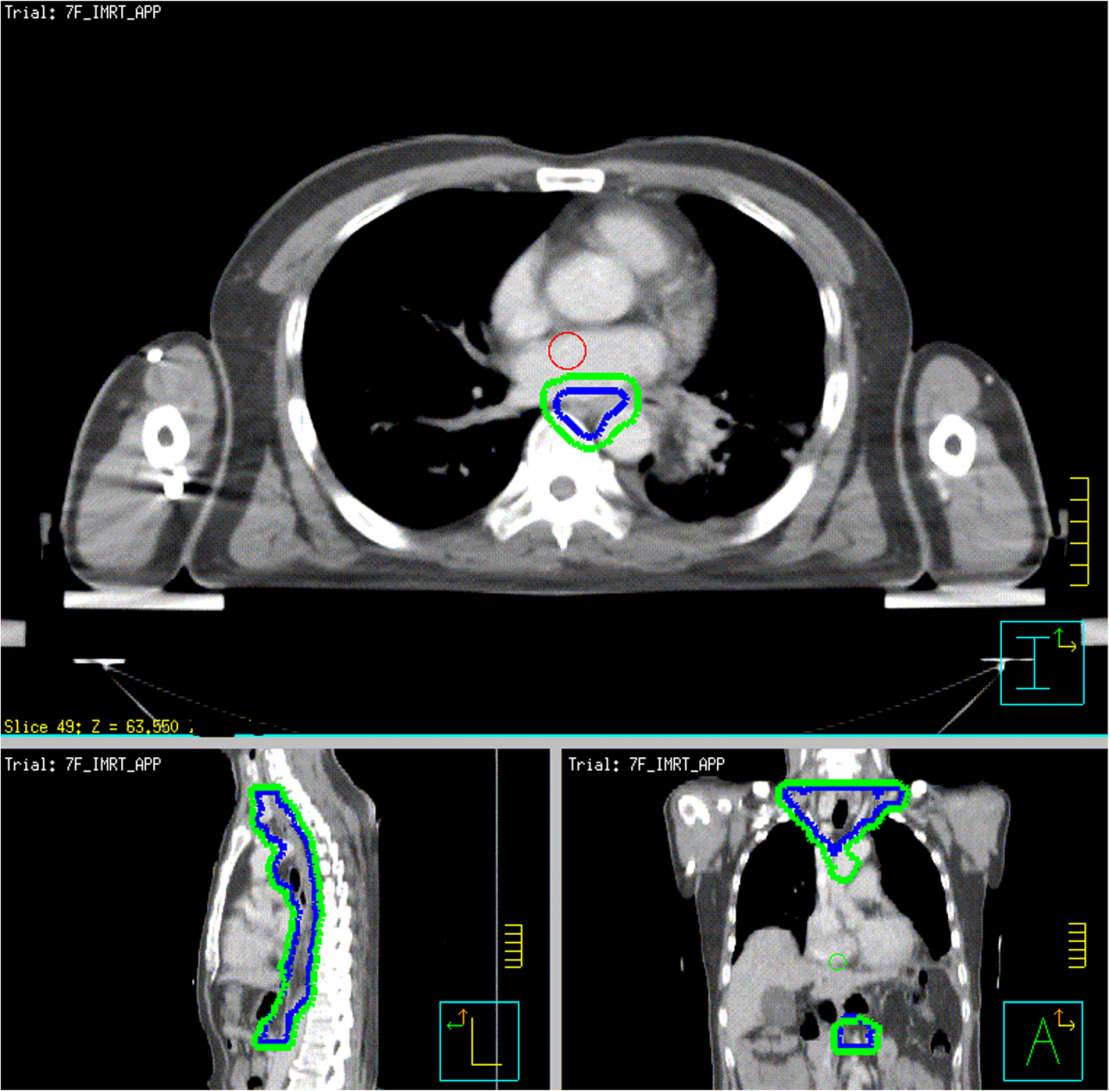
Lower-thoracic esophagus or middle-thoracic esophagus with metastasis in ≥3 regional lymph nodes distributed in two areas (mediastinal and under the diaphragm) or all in the subphrenic region (upper margin of the first thoracic vertebral body; lower margin: celiac axis; lymph-node stations include bilateral supraclavicular stations 1R, 1 L, 2R, 2 L, 3p, 4R, 4 L, 7, 8, 16, 17, 18, 19 and 20)

Treatment-planning CT using intravenous contrast will be undertaken for all patients in the supine position with arms straight beside the body. The clinical target volume (CTV) will be based on the tumor bed and corresponding lymphatic drainage areas. The planning target volume (PTV) will be generated using a uniform 0.5-cm expansion around the CTV. The contouring of the CTV for tumors in different locations are described in Figs. 1, 2 and 3. Anastomoses will be included in the CTV for patients with upper-thoracic tumors and patients who have an insufficient proximal margin (< 3 cm). The prescription dose will be 95% PTV 54 Gy/2.0 Gy/27 f.

#### POCRT

**Fig. 4:**
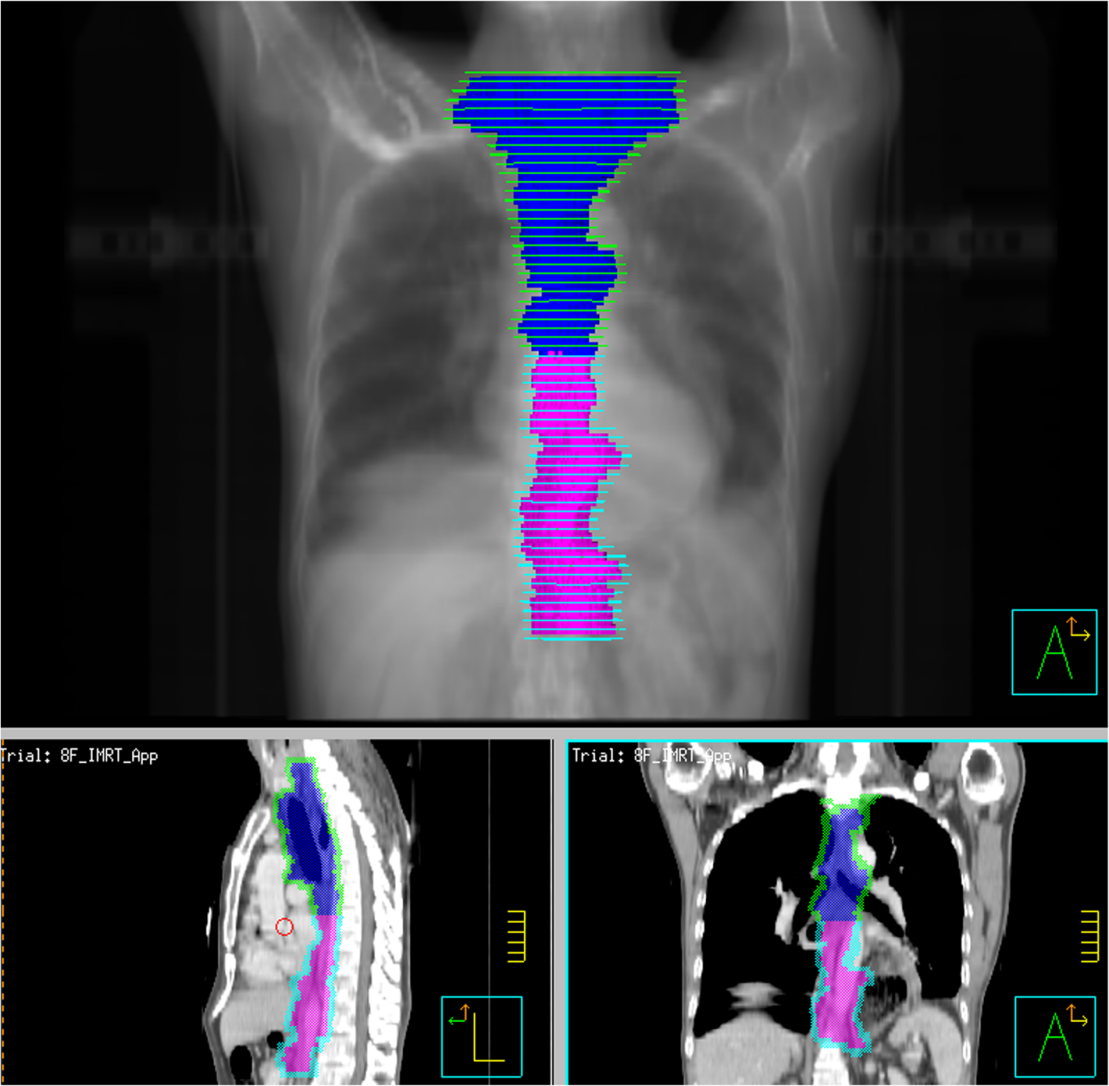
Target contouring for POCRT (Blue area is the CTV. Pink area is the omitted region)

The CTV borders will be defined superiorly as the cricothyroid membrane for upper-thoracic tumors or the upper margin of the first thoracic vertebral body for middle-thoracic tumors. The CTV borders will be defined inferiorly as 3-cm below the subcarina or the lower margin of the tumor bed (only for T4 lesions), including the lower cervical and bilateral supraclavicular region and mediastinal stations 1R/L, 2R/L, 3p, 4R/L, and 7 (Fig. 4). Anastomoses will be included in the CTV for patients with upper-thoracic tumors and patients who have an insufficient proximal margin (< 3 cm). The prescription dose will be 95% PTV 50.4 Gy/1.8 Gy/28 f.

Patients will receive paclitaxel (135–150 mg/m^2^) and cisplatin or nedaplatin (50–75) mg/m^2^ concurrent with RT. Injection with polyethylene glycol recombinant human granulocyte colony-stimulating factor for prophylaxis will be administered 48 h after chemotherapy. Chemotherapy will be repeated every 28 days for two courses in the absence of disease progression or unacceptable toxicity. Two or three cycles of consolidated chemotherapy can be undertaken in 1 month.

#### Constraints to organs at risk (OAR)

Lungs, heart, spinal cord, planning organ at risk volume (PRV) of the spinal cord and remnant stomach will be contoured on the simulation images. For lower thoracic esophageal cancer, the entire liver will be required to contouring. The volume of lung tissue receiving ≥20 Gy should not exceed 28% of the total lung volume (V20 < 28%). The mean dose of lung tissue should be lower than 17 Gy (D_mean_ lung < 17 Gy). Other dose constraints to OAR will be: V40 heart < 30%, V30 heart < 40%, D_max_ spinal cord PRV < 45 Gy, V40 remnant stomach < 40% without hot point, D_max_ remnant stomach < 55–60 Gy, V30 liver < 30%.

#### Surgery

The surgical approach and procedure will be based on tumor location. The Ivor Lewis esophagogastrectomy or McKeown esophagogastrectomy are the most common surgical approaches for upper-thoracic esophageal carcinomas. The Sweet esophagectomy is the most common surgical approach for middle- and lower-thoracic esophageal carcinomas. Video-assisted thoracoscopic surgery has been employed for esophagectomy in recent years. All patients will undergo R0 (defined as no cancer at resection margins).

### Toxicities and adverse events

Treatment-related toxicities and adverse events will be graded according to the toxicity criteria set by the Radiation Therapy Oncology Group and Common Terminology Criteria of Adverse Events v4.0. They will be recorded in detail on the case report forms of patients. The dosimetric parameters of OARs and the PTV should be recorded in detail. Serious adverse events will be reported to the Ethical Review Committee within 24 h and dealt with appropriately. Chemotherapy will be terminated in case of grade-4 hematogenous toxicity, grade-2 hepatic or renal dysfunction, grade-3 radiation pneumonitis and esophagitis, and other non-hematogenous grade-3 toxicities.

### Follow-up

Reexamination will be required every 3 months for the first 2 years, every 6 months for 3–5 years, and annually after 5 years. Physical examination and medical history will be documents: routine blood data; liver/kidney function; tumor markers, contrast-enhanced CT of the neck, chest and abdomen; ultrasonography of the neck and abdomen; esophagography; emission computed tomograph, CT or magnetic resonance imaging of the brain; cytologic puncture.

Tumor recurrence in regional lymph nodes will be defined according to UICC guidelines (7th edition), including supraclavicular, mediastinal, and abdominal lymph nodes (diaphragmatic, paracardial, left gastric, common hepatic, splenic, celiac). Sites of distant metastases are distant organs and non-regional lymph nodes. If the time interval between two recurrence sites is < 1 month, then this will be defined as “simultaneous recurrence”.

### Statistical analyses

DFS is defined as the period from surgery to time of the first recurrence and distant metastasis, death, or final follow-up. OS is defined as the interval from surgery to death from any cause or final follow-up.

Intention-to-treat and per-protocol set analyses will be adopted. Statistical analyses will be carried out using SPSS v20.0 (IBM, Armonk, NY, USA). *p* < 0.05 (two-tailed) will be used to denote a significant difference. The Kaplan–Meier method will be used to calculate DFS and OS. The log-rank method will be employed to determine the significance. COX multivariate analysis will be conducted to identify independent prognostic factors.

### Ethical considerations

The study protocol has been approved by the ethics committee of the Chinese Academy of Medical Sciences (14–090/880). Written informed consent will be obtained before enrollment. The study has been registered in ClinicalTrails.gov. (NCT02279134).

## Discussion

National Comprehensive Cancer Network guidelines (2016–2019) recommend observation for pathologic T1-4aN0-1 M0 esophageal squamous cell carcinomas. However, 5-year OS decreases from 60.0 to 18.0% with increasing stage from IIA to III (*p* < 0.001) after surgery. Postoperative recurrence rates also vary with the number of lymph-node metastases (16, 44, 69 and 93% according to 0, 1–2, 3–7 and > 8, respectively p < 0.001) [24]. Also, recurrence in regional lymph nodes is predominant. The median time of recurrence is about 10.0–17.0 months [5, 6, 8, 25, 26]. Hence, PORT is indispensable for patients receiving SA. However, only a few small-sample-size studies on PORT by conventional radiotherapy methods have been carried out, and have shown that PORT does not improve OS. Besides, the irradiation field and PORT dose for esophageal cancer after surgery are controversial [10, 12, 13, 27–29]. The main reasons are that studies enrolled patients with different: pathologic tumor stages; status of lymph-node metastases; sites of esophageal cancer. This led to different recurrence patterns. Thus, obtaining consistent results without subgroup analyses is challenging. Therefore, a stratified study to identify patients who may benefit from adjuvant treatment is warranted urgently.

Xiao and colleagues [13, 30] reported that PORT can reduce the recurrence rate at radiotherapy sites and improve OS in patients with pathologic stage-III and lymph node-positive esophageal carcinomas. Although OS did not differ significantly between the SA group and PORT for stage-IIA or lymph node-negative cases, 3- year OS increased by approximately 8.0–10.0%. Therefore, according to the recurrence rate and failure patterns of esophageal cancer after surgery, Xiao and coworkers designed different irradiation fields according to different locations of esophageal carcinoma and different lymph-node status.

With the development of radiotherapy methods, three dimensional-conformal radiation therapy and IMRT have been used widely. A prospective study on postoperative irradiation fields employed from 2004 to 2009 in our institution was launched to analyze tumor recurrence patterns after surgery. Results showed that, for pathologic lymph node-positive or stage-III patients, postoperative IMRT could further improve OS (*p* < 0.05) and reduce the recurrence rate in mediastinal lymph nodes from 34.6 to 13.4% (*p* < 0.001) and in supraclavicular lymph nodes from 13.3 to 6.1% (*p* = 0.015). There was no significant difference in recurrence in abdominal lymph nodes between the two groups (9.8 and 7.8%, respectively, *p* = 0.510). The rate of hematogenous metastasis in the PORT group (30.7%) was higher than that in the SA group (21.0%, *p* = 0.020) [20]. That study provided an important basis for POCRT of esophageal squamous cell carcinoma.

POCRT for esophageal adenocarcinomas or adenocarcinomas at the esophagogastric junction can improve OS in pT3–4 or lymph node-positive patients [27, 31–33]. However, few retrospective studies have shown that POCRT can improve OS in patients with lymph node-positive or stage-III TESCC [21–23]. Therefore, a prospective study on the irradiation field, irradiation dose, and dose of chemotherapy for POCRT is needed.

A phase-I study has been carried out to determine the optimal irradiation field and optimal dose for patients with positive lymph nodes and stage-III TESCC combined with concurrent chemotherapy in our institution. The irradiation field is contoured from the first thoracic vertebral body to the celiac axis at 54 Gy or 60 Gy. Concurrent five weekly cycles of chemotherapy are expected. Although grade-5 toxicity did not occur, escalation of the chemotherapy dose failed due to dose-limiting toxicity at the beginning of treatment. This might be one of the reasons why it is difficult to carry out a study of concurrent POCRT. Hence, the irradiation dose and irradiation field must be adjusted rationally. Due to the difficult anatomy and surgical field, recent studies have shown that supraclavicular and mediastinal lymph nodes are the most common recurrence regions after surgery [5, 34–36]. These regions are always included in the irradiation region for PORT. Therefore, considering the low recurrence rate in abdominal lymph nodes (2.1–10.4%) [5, 37–40], a phase-I study by rational reduction of the irradiation field (omitting the abdominal lymphatic drainage area) was carried out again. It showed that IMRT with small-target, concurrent five weekly cycles of chemotherapy were safe and efficacious in patients with positive lymph nodes and stage-III disease after surgery. Only one patient had recurrence in abdominal lymph nodes after reducing the irradiation area, and the recurrence site was below the abdominal lymphatic drainage area [41]. Then, a phase-II study was conducted using this scheme and 65 patients enrolled (unpublished). As many as 69.2% of patients (45/65 patients) did not complete the full dose of CRT due to refusal or intolerance. Therefore, on the basis of the phase-I/II study, a phase-II/III prospective RCT must be carried out to validate safety and efficacy by further reducing the irradiation dose to 50.4 Gy and reducing the irradiation field appropriately to guarantee completion of concurrent CRT. Primarily, this RCT will determine whether POCRT can further improve local control and decrease hematogenous metastasis compared with PORT, and whether it can continue to improve OS compared with SA.

## Data Availability

Not applicable – data collection is still ongoing.

## Abbreviations

CRT: Chemoradiotherapy
CT: Computed tomography
CTV: Clinical target volume
DFS: Disease-free survival
IMRT: Intensity-modulated radiotherapy
OAR: Organs at risk
OoFRRR: Out-of-field regional recurrence rate
OS: Overall survival
POCRT: Postoperative chemoradiotherapy
PORT: Postoperative radiotherapy
PRV: Planning organ at risk volume
PTV: Planning target volume
RCT: Randomized controlled trial
SA: Surgery alone
TESCC: Thoracic esophageal squamous cell carcinoma
UICC: Union for International Cancer Control
